# 3-(4-Chloro­benzene­sulfonamido)-5-methyl­cyclo­hex-2-en-1-one

**DOI:** 10.1107/S1600536811030662

**Published:** 2011-08-06

**Authors:** Patrice L. Jackson, Henry North, Mariano S. Alexander, Gervas E. Assey, Kenneth R. Scott, Ray J. Butcher

**Affiliations:** a College of Pharmacy, University of Maryland Eastern Shore, Princess Anne, MD 21853, USA; b Department of Pharmaceutical Sciences, Howard University, 2300 4th Street NW, Washington, DC 20059, USA; c Department of Chemistry, Howard University, 525 College Street NW, Washington, DC 20059, USA

## Abstract

For the title compound, C_13_H_14_ClNO_3_S, geometrical parameters, determined using X-ray diffraction techniques, are compared with those calculated by density functional theory (DFT), using hybrid exchange-correlation functional, B3LYP methods. The dihedral angle between the benzene ring and the conjugated part of the cyclo­hexene ring is 87.47 (5)°. The cyclo­hexene ring and its substituents are disordered over two conformations, with occupancies of 0.786 (3) and 0.214 (3). In the crystal, mol­ecules are linked into chains in the *c*-axis direction by inter­molecular N—H⋯O(C=O) hydrogen bonds. C—H⋯O inter­actions are also observed.

## Related literature

For the crystal growth of the title compound, see: Assey (2010 ▶). For related enaminone structures and properties, see: Edafiogho *et al.* (2006 ▶, 2007 ▶); Eddington *et al.* (2000 ▶); Jackson (2009 ▶); Michael *et al.* (1996 ▶, 2001 ▶). For their anti-convulsant activity, see: Stables & Kupferburg (1997 ▶). For information related to *GAUSSIAN* software, see: Frisch *et al.* (2004 ▶)
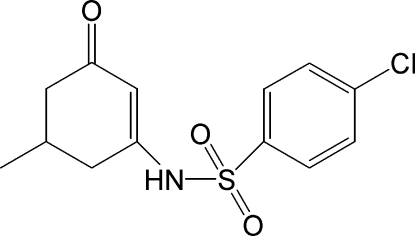


## Experimental

### 

#### Crystal data

C_13_H_14_ClNO_3_S
*M* *_r_* = 299.76
Monoclinic, 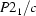

*a* = 10.2031 (2) Å
*b* = 10.3267 (3) Å
*c* = 14.1217 (3) Å
β = 108.989 (3)°
*V* = 1406.95 (6) Å^3^
*Z* = 4
Cu *K*α radiation
μ = 3.83 mm^−1^
*T* = 295 K
0.76 × 0.61 × 0.31 mm

#### Data collection

Oxford Diffraction Gemini R diffractometer
Absorption correction: analytical [*CrysAlis RED* (Oxford Diffraction, 2009 ▶), based on expressions derived by Clark & Reid (1995 ▶)] *T*_min_ = 0.119, *T*_max_ = 0.355
5137 measured reflections
2778 independent reflections
2547 reflections with *I* > 2σ(*I*)
*R*_int_ = 0.047

#### Refinement

*R*[*F* ^2^ > 2σ(*F* ^2^)] = 0.083
*wR*(*F* ^2^) = 0.250
*S* = 1.07
2778 reflections
205 parameters
18 restraints
H atoms treated by a mixture of independent and constrained refinement
Δρ_max_ = 1.26 e Å^−3^
Δρ_min_ = −0.65 e Å^−3^

### 

Data collection: *CrysAlis CCD* (Oxford Diffraction, 2009 ▶); cell refinement: *CrysAlis RED* (Oxford Diffraction, 2009 ▶); data reduction: *CrysAlis RED*; program(s) used to solve structure: *SHELXS97* (Sheldrick, 2008 ▶); program(s) used to refine structure: *SHELXL97* (Sheldrick, 2008 ▶); molecular graphics: *SHELXTL* (Sheldrick, 2008 ▶); software used to prepare material for publication: *SHELXTL*.

## Supplementary Material

Crystal structure: contains datablock(s) I, global. DOI: 10.1107/S1600536811030662/hg5071sup1.cif

Structure factors: contains datablock(s) I. DOI: 10.1107/S1600536811030662/hg5071Isup2.hkl

Supplementary material file. DOI: 10.1107/S1600536811030662/hg5071Isup3.cml

Additional supplementary materials:  crystallographic information; 3D view; checkCIF report

## Figures and Tables

**Table 1:** Hydrogen-bond geometry (Å, °)

*D*—H⋯*A*	*D*—H	H⋯*A*	*D*⋯*A*	*D*—H⋯*A*
N—H1*N*⋯O3*B*′^i^	0.83 (2)	1.91 (2)	2.729 (10)	166 (2)
N—H1*N*⋯O3*A*′^i^	0.83 (2)	1.95 (2)	2.777 (3)	171 (2)
C5—H5*A*⋯O2^ii^	0.93	2.53	3.337 (3)	145

Symmetry codes: (i) 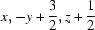; (ii) 
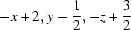
.

**Fig. 1.**
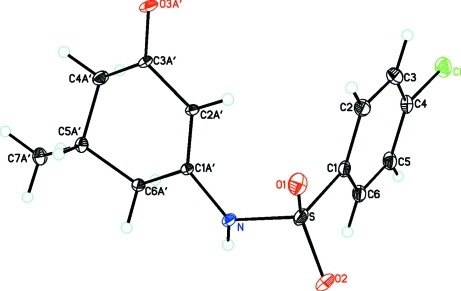
Diagram of 3-[(4'-chloro)benzenesulfonylamino]-5-methylcyclohex-2-enone showing the major component. Thermal ellipsoids drawn at the 30% probability level.

**Fig. 2.**
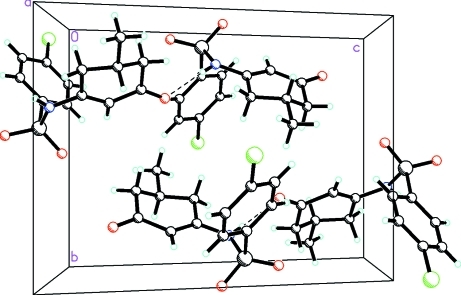
The molecular packing for 3-[(4'-chloro)benzenesulfonylamino]-5-methylcyclohex-2-enone viewed down the a axis. Intermolecular interactions are shown by dashed lines.

## supplementary crystallographic information

### Comment

Enaminone analogues are reported to possess antiflammatory (Eddington *et al.*, 2000) antimalarial (Edafiogho *et al.*, 2006), antibacterial (Michael *et al.*, 1996, 2001), and anticonvulsant properties (Edafiogho *et al.*, 2007). Over recent years studies have shown that enaminones and their derivatives have played a major role in anti-epileptic activity and a number of structurally diverse anticonvulsant active enaminone analogues have been synthesized in our laboratory. We have provided several enaminones and their derivatives that are highly active in anticonvulsant studies. Our recent research has produced a novel series of benzene sulfonamide enaminones that are active in anticonvulsant studies. One of the compounds, [3-[(4'-chloro)benzenesulfonylamino]-5-methylcyclohex-2-enone, has been studied by pharmacology, X-ray and DFT studies (Jackson, 2009; Assey, 2010). Pharmacology was performed at the National Institute of Neurological Disorders and Stroke (NINDS), National Institute of Health (NIH) (Stables & Kupferburg, 1997). Density-functional theory (DFT) and Hartree-Fock calculations and full-geometry optimizations were performed by means of the *GAUSSIAN* 03 W package (Frisch, *et al.*, 2004). The selected bond lengths and angles obtained from HF and DFT/B3LYP are given in Table 2.

The structure of the title compound, 3-[(4'-chloro)benzenesulfonylamino]-5-methylcyclohex-2-enone, C_13_H_14_ClNO_3_S, the shape of the molecule is important in determining binding to the receptor sites thus it is of interest to note that the dihedral angle between the phenyl ring and the conjugated part of the cyclohexene ring is 87.47 (5)°. The cyclohexene and its substituents are disordered over two conformations with occupancies of 0.786 (3) and 0.214 (3), respectively. The molecules are linked in chains in the c direction by intermolecular N—H···O(C=O) hydrogen bonds.

### Experimental

3-amino-5-methylcyclohex-2-enone (2.00 g, 16 mmol) and NaH (1.07 g, 44.8 mmol) in dry THF refluxed for 1 h. After cooling, a solution of *p*-chlorobenzenesulfonyl chloride (3.42 g, 16.2 mmol) in 30 ml of dry THF was added dropwise. The reaction mixture was allowed to reflux for 1 h. Upon workup, the mixture is cooled to room temperature and quenched with 150 ml of deionized H_2_O and acidified with 12 ml of concentrated HCl. The aqueous solution was extracted with dichloromethane (2 *x* 100 ml) and the organic layer washed with 80 ml of water. The organic phase was dried over MgSO_4_, filtered, and evaporated *in vacuo* at a temperature not exceeding 35°C (32% yield) (1.54 g), as a white powder from methylene chloride, mp 191–193°C. νmax (cm^-1^) = νNH 3093 (w); ν*sp*^2^ 3031 (CH stretch; w); νC=O 1615 (*m*); νC=C 1609 and 1476 (aromatic); νS=O 1332(asymmetric; m) and 1141 (symmetric; m); νCN 1164 (s, sh); and νCl-aryl 1088 (m, sh). ^1^H NMR: δ (DMSO-d_6_) 0.915 (3*H*, d, CH3); 1.86–2.40 (5*H*, m, cyclohexene ring); 5.54 (1*H*, s, =CH); 7.76–7.86 (4*H*, dd, aromatic ring); 10.90 (1*H*, s, NH). Anal. Calculated for C^13^H^14^ClN^2^O^5^S: C, 52.09; H, 4.71; N, 4.67; S, 10.70. Found: C, 52.03; H, 4.59; N, 4.30; S, 10.67. Crystals of 3-[(4'-chloro)benzenesulfonylamino]-5-methylcyclohex-2-enone were obtained by slow evaporation from acetonitrile.

### Refinement

H atoms were placed in geometrically idealized positions and constrained to ride on their parent atoms with a C—H distance between 0.93 and 0.98 Å *U*_iso_(H) = 1.2*U*_eq_(C) and 0.96 Å for CH_3_ [*U*_iso_(H) = 1.5*U*_eq_(C)]. The H atom attached to N was refined isotropically. The the cyclohexene ring and its substituents were disordered over two conformations with occupancies of 0.786 (3) and 0.214 (3), respectively.

### Crystal data

**Table:**

C_13_H_14_ClNO_3_S	*F*(000) = 624
*M**_r_* = 299.76	*D*_x_ = 1.415 Mg m^−^^3^
Monoclinic, *P*2_1_/*c*	Cu *K*α radiation, λ = 1.54184 Å
Hall symbol: -P 2ybc	Cell parameters from 4047 reflections
*a* = 10.2031 (2) Å	θ = 4.3–77.2°
*b* = 10.3267 (3) Å	µ = 3.83 mm^−^^1^
*c* = 14.1217 (3) Å	*T* = 295 K
β = 108.989 (3)°	Large plate, colorless
*V* = 1406.95 (6) Å^3^	0.76 × 0.61 × 0.31 mm
*Z* = 4	

### Data collection

**Table:**

Oxford Diffraction Gemini R diffractometer	2778 independent reflections
Radiation source: fine-focus sealed tube	2547 reflections with *I* > 2σ(*I*)
graphite	*R*_int_ = 0.047
Detector resolution: 10.5081 pixels mm^-1^	θ_max_ = 77.7°, θ_min_ = 4.6°
φ and ω scans	*h* = −12→11
Absorption correction: analytical [*CrysAlis RED* (Oxford Diffraction, 2009), based on expressions derived by Clark & Reid (1995)]	*k* = −11→12
*T*_min_ = 0.119, *T*_max_ = 0.355	*l* = −17→16
5137 measured reflections	

### Refinement

**Table:**

Refinement on *F*^2^	Secondary atom site location: difference Fourier map
Least-squares matrix: full	Hydrogen site location: inferred from neighbouring sites
*R*[*F*^2^ > 2σ(*F*^2^)] = 0.083	H atoms treated by a mixture of independent and constrained refinement
*wR*(*F*^2^) = 0.250	*w* = 1/[σ^2^(*F*_o_^2^) + (0.183*P*)^2^ + 0.6772*P*] where *P* = (*F*_o_^2^ + 2*F*_c_^2^)/3
*S* = 1.07	(Δ/σ)_max_ = 0.001
2778 reflections	Δρ_max_ = 1.26 e Å^−^^3^
205 parameters	Δρ_min_ = −0.65 e Å^−^^3^
18 restraints	Extinction correction: *SHELXL97* (Sheldrick, 2008), Fc^*^=kFc[1+0.001xFc^2^λ^3^/sin(2θ)]^-1/4^
Primary atom site location: structure-invariant direct methods	Extinction coefficient: 0.0074 (13)

### Special details

**Table:**

Experimental. CrysAlis RED (Oxford Diffraction , 2009) Analytical numeric absorption correction using a multifaceted crystal model based on expressions derived by R.C. Clark & J.S. Reid. (Clark, R. C. & Reid, J. S. (1995). Acta Cryst. A51, 887-897)
Geometry. All e.s.d.'s (except the e.s.d. in the dihedral angle between two l.s. planes) are estimated using the full covariance matrix. The cell e.s.d.'s are taken into account individually in the estimation of e.s.d.'s in distances, angles and torsion angles; correlations between e.s.d.'s in cell parameters are only used when they are defined by crystal symmetry. An approximate (isotropic) treatment of cell e.s.d.'s is used for estimating e.s.d.'s involving l.s. planes.
Refinement. Refinement of *F*^2^ against ALL reflections. The weighted *R*-factor *wR* and goodness of fit *S* are based on *F*^2^, conventional *R*-factors *R* are based on *F*, with *F* set to zero for negative *F*^2^. The threshold expression of *F*^2^ > σ(*F*^2^) is used only for calculating *R*-factors(gt) *etc*. and is not relevant to the choice of reflections for refinement. *R*-factors based on *F*^2^ are statistically about twice as large as those based on *F*, and *R*- factors based on ALL data will be even larger.

### Fractional atomic coordinates and isotropic or equivalent isotropic displacement parameters (Å^2^)

**Table:**

	*x*	*y*	*z*	*U*_iso_*/*U*_eq_	Occ. (<1)
Cl	1.22572 (8)	0.52485 (10)	0.58620 (7)	0.0804 (3)	
S	0.75611 (6)	0.92036 (5)	0.56637 (4)	0.04691 (16)	
O1	0.7602 (2)	1.02174 (17)	0.49885 (16)	0.0609 (5)	
O2	0.7649 (2)	0.9502 (2)	0.66719 (15)	0.0706 (6)	
N	0.60950 (18)	0.84131 (18)	0.52132 (10)	0.0412 (4)	
H1N	0.591 (2)	0.802 (2)	0.5668 (14)	0.048 (7)*	
C1	0.8877 (2)	0.8073 (2)	0.56969 (17)	0.0441 (5)	
C2	0.9599 (3)	0.8170 (3)	0.5024 (2)	0.0538 (6)	
H2A	0.9385	0.8817	0.4540	0.065*	
C3	1.0638 (3)	0.7296 (3)	0.5081 (2)	0.0605 (7)	
H3A	1.1137	0.7351	0.4636	0.073*	
C4	1.0935 (2)	0.6346 (3)	0.5792 (2)	0.0557 (7)	
C5	1.0217 (3)	0.6238 (3)	0.6468 (2)	0.0596 (7)	
H5A	1.0433	0.5585	0.6948	0.072*	
C6	0.9181 (2)	0.7110 (3)	0.64169 (19)	0.0534 (6)	
H6A	0.8686	0.7054	0.6864	0.064*	
O3A'	0.5775 (3)	0.8028 (3)	0.17833 (16)	0.0530 (5)	0.786 (3)
C1A'	0.5533 (3)	0.7962 (2)	0.42417 (12)	0.0339 (4)	0.786 (3)
C2A'	0.6036 (3)	0.8212 (3)	0.34828 (19)	0.0364 (5)	0.786 (3)
H2AA	0.6808	0.8745	0.3599	0.044*	0.786 (3)
C3A'	0.5401 (3)	0.7671 (3)	0.2502 (2)	0.0393 (5)	0.786 (3)
C4A'	0.4276 (3)	0.6688 (3)	0.2359 (2)	0.0466 (7)	0.786 (3)
H4AA	0.4685	0.5832	0.2497	0.056*	0.786 (3)
H4AB	0.3677	0.6704	0.1666	0.056*	0.786 (3)
C5A'	0.3411 (3)	0.6942 (3)	0.3040 (2)	0.0437 (7)	0.786 (3)
H5AA	0.2954	0.7784	0.2855	0.052*	0.786 (3)
C6A'	0.4363 (3)	0.7032 (3)	0.4122 (2)	0.0421 (6)	0.786 (3)
H6AA	0.3828	0.7308	0.4542	0.051*	0.786 (3)
H6AB	0.4738	0.6181	0.4347	0.051*	0.786 (3)
C7A'	0.2296 (4)	0.5931 (4)	0.2902 (3)	0.0558 (9)	0.786 (3)
H7AA	0.1709	0.5922	0.2215	0.084*	0.786 (3)
H7AB	0.1753	0.6132	0.3324	0.084*	0.786 (3)
H7AC	0.2718	0.5096	0.3079	0.084*	0.786 (3)
O3B'	0.5921 (10)	0.7747 (12)	0.1904 (6)	0.0530 (5)	0.214 (3)
C1B'	0.5549 (9)	0.7864 (5)	0.4272 (2)	0.0339 (4)	0.214 (3)
C2B'	0.5841 (11)	0.8367 (12)	0.3478 (6)	0.0364 (5)	0.214 (3)
H2BA	0.6405	0.9093	0.3552	0.044*	0.214 (3)
C3B'	0.5260 (10)	0.7750 (12)	0.2512 (7)	0.0393 (5)	0.214 (3)
C4B'	0.3941 (10)	0.7012 (12)	0.2334 (7)	0.0466 (7)	0.214 (3)
H4BA	0.3842	0.6399	0.1795	0.056*	0.214 (3)
H4BB	0.3169	0.7612	0.2119	0.056*	0.214 (3)
C5B'	0.3867 (10)	0.6284 (10)	0.3246 (6)	0.0437 (7)	0.214 (3)
H5BA	0.4570	0.5600	0.3395	0.052*	0.214 (3)
C6B'	0.4207 (11)	0.7179 (13)	0.4147 (8)	0.0421 (6)	0.214 (3)
H6BA	0.3470	0.7808	0.4053	0.051*	0.214 (3)
H6BB	0.4282	0.6680	0.4744	0.051*	0.214 (3)
C7B'	0.2415 (16)	0.5623 (17)	0.3043 (14)	0.0558 (9)	0.214 (3)
H7BA	0.1711	0.6277	0.2920	0.084*	0.214 (3)
H7BB	0.2423	0.5120	0.3617	0.084*	0.214 (3)
H7BC	0.2225	0.5068	0.2469	0.084*	0.214 (3)

### Atomic displacement parameters (Å^2^)

**Table:**

	*U*^11^	*U*^22^	*U*^33^	*U*^12^	*U*^13^	*U*^23^
Cl	0.0536 (4)	0.0905 (5)	0.0885 (6)	0.0121 (3)	0.0112 (4)	−0.0009 (4)
S	0.0462 (3)	0.0526 (3)	0.0352 (3)	−0.0116 (2)	0.0040 (2)	−0.00969 (19)
O1	0.0640 (10)	0.0465 (9)	0.0670 (11)	−0.0130 (8)	0.0142 (8)	0.0008 (8)
O2	0.0764 (12)	0.0880 (12)	0.0396 (9)	−0.0123 (11)	0.0081 (8)	−0.0268 (9)
N	0.0399 (8)	0.0581 (10)	0.0253 (7)	−0.0102 (7)	0.0103 (6)	−0.0050 (7)
C1	0.0329 (9)	0.0557 (11)	0.0368 (10)	−0.0111 (8)	0.0019 (8)	0.0004 (8)
C2	0.0531 (11)	0.0599 (13)	0.0486 (12)	−0.0120 (10)	0.0170 (10)	0.0062 (10)
C3	0.0536 (12)	0.0721 (16)	0.0600 (14)	−0.0106 (12)	0.0243 (10)	0.0014 (12)
C4	0.0339 (10)	0.0649 (14)	0.0592 (14)	−0.0056 (10)	0.0026 (9)	−0.0020 (12)
C5	0.0433 (11)	0.0763 (16)	0.0511 (13)	−0.0075 (11)	0.0044 (10)	0.0162 (12)
C6	0.0404 (10)	0.0725 (15)	0.0426 (11)	−0.0100 (10)	0.0070 (9)	0.0114 (10)
O3A'	0.0683 (9)	0.0686 (13)	0.0278 (8)	−0.0041 (9)	0.0235 (7)	0.0011 (7)
C1A'	0.0317 (8)	0.0430 (9)	0.0264 (8)	−0.0005 (7)	0.0083 (6)	0.0000 (7)
C2A'	0.0329 (9)	0.0498 (11)	0.0272 (9)	−0.0061 (9)	0.0108 (7)	−0.0035 (8)
C3A'	0.0405 (10)	0.0513 (11)	0.0274 (9)	0.0008 (9)	0.0129 (8)	−0.0024 (8)
C4A'	0.0476 (14)	0.0558 (16)	0.0347 (11)	−0.0052 (12)	0.0112 (10)	−0.0115 (10)
C5A'	0.0333 (11)	0.0538 (15)	0.0403 (13)	−0.0072 (10)	0.0071 (9)	−0.0063 (11)
C6A'	0.0367 (10)	0.0599 (13)	0.0313 (9)	−0.0072 (9)	0.0132 (8)	0.0001 (9)
C7A'	0.0404 (11)	0.0595 (19)	0.0639 (17)	−0.0150 (12)	0.0122 (11)	−0.0074 (14)
O3B'	0.0683 (9)	0.0686 (13)	0.0278 (8)	−0.0041 (9)	0.0235 (7)	0.0011 (7)
C1B'	0.0317 (8)	0.0430 (9)	0.0264 (8)	−0.0005 (7)	0.0083 (6)	0.0000 (7)
C2B'	0.0329 (9)	0.0498 (11)	0.0272 (9)	−0.0061 (9)	0.0108 (7)	−0.0035 (8)
C3B'	0.0405 (10)	0.0513 (11)	0.0274 (9)	0.0008 (9)	0.0129 (8)	−0.0024 (8)
C4B'	0.0476 (14)	0.0558 (16)	0.0347 (11)	−0.0052 (12)	0.0112 (10)	−0.0115 (10)
C5B'	0.0333 (11)	0.0538 (15)	0.0403 (13)	−0.0072 (10)	0.0071 (9)	−0.0063 (11)
C6B'	0.0367 (10)	0.0599 (13)	0.0313 (9)	−0.0072 (9)	0.0132 (8)	0.0001 (9)
C7B'	0.0404 (11)	0.0595 (19)	0.0639 (17)	−0.0150 (12)	0.0122 (11)	−0.0074 (14)

### Geometric parameters (Å, °)

**Table:**

Cl—C4	1.740 (3)	C4A'—H4AB	0.9700
S—O1	1.426 (2)	C5A'—C7A'	1.509 (5)
S—O2	1.431 (2)	C5A'—C6A'	1.524 (4)
S—N	1.6400 (17)	C5A'—H5AA	0.9800
S—C1	1.768 (2)	C6A'—H6AA	0.9700
N—C1B'	1.384 (2)	C6A'—H6AB	0.9700
N—C1A'	1.3843 (18)	C7A'—H7AA	0.9600
N—H1N	0.833 (16)	C7A'—H7AB	0.9600
C1—C2	1.382 (4)	C7A'—H7AC	0.9600
C1—C6	1.383 (3)	O3B'—C3B'	1.252 (12)
C2—C3	1.375 (4)	C1B'—C2B'	1.355 (10)
C2—H2A	0.9300	C1B'—C6B'	1.499 (11)
C3—C4	1.365 (4)	C2B'—C3B'	1.446 (11)
C3—H3A	0.9300	C2B'—H2BA	0.9300
C4—C5	1.384 (4)	C3B'—C4B'	1.494 (12)
C5—C6	1.373 (4)	C4B'—C5B'	1.514 (12)
C5—H5A	0.9300	C4B'—H4BA	0.9700
C6—H6A	0.9300	C4B'—H4BB	0.9700
O3A'—C3A'	1.251 (4)	C5B'—C6B'	1.519 (12)
C1A'—C2A'	1.356 (4)	C5B'—C7B'	1.570 (18)
C1A'—C6A'	1.498 (4)	C5B'—H5BA	0.9800
C2A'—C3A'	1.437 (3)	C6B'—H6BA	0.9700
C2A'—H2AA	0.9300	C6B'—H6BB	0.9700
C3A'—C4A'	1.496 (4)	C7B'—H7BA	0.9600
C4A'—C5A'	1.525 (4)	C7B'—H7BB	0.9600
C4A'—H4AA	0.9700	C7B'—H7BC	0.9600
			
O1—S—O2	120.11 (14)	C7A'—C5A'—C4A'	111.5 (3)
O1—S—N	109.11 (10)	C6A'—C5A'—C4A'	109.4 (2)
O2—S—N	104.28 (12)	C7A'—C5A'—H5AA	107.8
O1—S—C1	108.39 (12)	C6A'—C5A'—H5AA	107.8
O2—S—C1	108.34 (13)	C4A'—C5A'—H5AA	107.8
N—S—C1	105.70 (10)	C1A'—C6A'—C5A'	112.1 (2)
C1B'—N—S	127.4 (4)	C1A'—C6A'—H6AA	109.2
C1A'—N—S	125.70 (16)	C5A'—C6A'—H6AA	109.2
C1B'—N—H1N	114.7 (17)	C1A'—C6A'—H6AB	109.2
C1A'—N—H1N	118.3 (16)	C5A'—C6A'—H6AB	109.2
S—N—H1N	110.6 (15)	H6AA—C6A'—H6AB	107.9
C2—C1—C6	121.0 (2)	C2B'—C1B'—N	120.6 (6)
C2—C1—S	120.28 (19)	C2B'—C1B'—C6B'	121.5 (6)
C6—C1—S	118.7 (2)	N—C1B'—C6B'	112.0 (6)
C3—C2—C1	119.1 (2)	C1B'—C2B'—C3B'	118.6 (9)
C3—C2—H2A	120.5	C1B'—C2B'—H2BA	120.7
C1—C2—H2A	120.5	C3B'—C2B'—H2BA	120.7
C4—C3—C2	119.8 (3)	O3B'—C3B'—C2B'	120.0 (9)
C4—C3—H3A	120.1	O3B'—C3B'—C4B'	122.7 (9)
C2—C3—H3A	120.1	C2B'—C3B'—C4B'	117.0 (9)
C3—C4—C5	121.7 (3)	C3B'—C4B'—C5B'	113.7 (8)
C3—C4—Cl	119.5 (2)	C3B'—C4B'—H4BA	108.8
C5—C4—Cl	118.9 (2)	C5B'—C4B'—H4BA	108.8
C6—C5—C4	118.8 (3)	C3B'—C4B'—H4BB	108.8
C6—C5—H5A	120.6	C5B'—C4B'—H4BB	108.8
C4—C5—H5A	120.6	H4BA—C4B'—H4BB	107.7
C5—C6—C1	119.7 (3)	C4B'—C5B'—C6B'	110.5 (9)
C5—C6—H6A	120.2	C4B'—C5B'—C7B'	111.5 (9)
C1—C6—H6A	120.2	C6B'—C5B'—C7B'	111.2 (10)
C2A'—C1A'—N	125.3 (2)	C4B'—C5B'—H5BA	107.8
C2A'—C1A'—C6A'	121.7 (2)	C6B'—C5B'—H5BA	107.8
N—C1A'—C6A'	112.7 (2)	C7B'—C5B'—H5BA	107.8
C1A'—C2A'—C3A'	121.3 (2)	C1B'—C6B'—C5B'	109.7 (8)
C1A'—C2A'—H2AA	119.4	C1B'—C6B'—H6BA	109.7
C3A'—C2A'—H2AA	119.4	C5B'—C6B'—H6BA	109.7
O3A'—C3A'—C2A'	120.4 (3)	C1B'—C6B'—H6BB	109.7
O3A'—C3A'—C4A'	120.9 (2)	C5B'—C6B'—H6BB	109.7
C2A'—C3A'—C4A'	118.7 (3)	H6BA—C6B'—H6BB	108.2
C3A'—C4A'—C5A'	111.9 (2)	C5B'—C7B'—H7BA	109.5
C3A'—C4A'—H4AA	109.2	C5B'—C7B'—H7BB	109.5
C5A'—C4A'—H4AA	109.2	H7BA—C7B'—H7BB	109.5
C3A'—C4A'—H4AB	109.2	C5B'—C7B'—H7BC	109.5
C5A'—C4A'—H4AB	109.2	H7BA—C7B'—H7BC	109.5
H4AA—C4A'—H4AB	107.9	H7BB—C7B'—H7BC	109.5
C7A'—C5A'—C6A'	112.3 (3)		
			
O1—S—N—C1B'	53.1 (4)	C6A'—C1A'—C2A'—C3A'	4.8 (4)
O2—S—N—C1B'	−177.4 (4)	C1A'—C2A'—C3A'—O3A'	171.7 (3)
C1—S—N—C1B'	−63.2 (4)	C1A'—C2A'—C3A'—C4A'	−7.5 (4)
O1—S—N—C1A'	48.0 (2)	O3A'—C3A'—C4A'—C5A'	−146.2 (3)
O2—S—N—C1A'	177.5 (2)	C2A'—C3A'—C4A'—C5A'	33.0 (4)
C1—S—N—C1A'	−68.4 (2)	C3A'—C4A'—C5A'—C7A'	−178.9 (3)
O1—S—C1—C2	−9.0 (2)	C3A'—C4A'—C5A'—C6A'	−54.2 (3)
O2—S—C1—C2	−140.8 (2)	C2A'—C1A'—C6A'—C5A'	−27.7 (4)
N—S—C1—C2	107.90 (19)	N—C1A'—C6A'—C5A'	158.0 (2)
O1—S—C1—C6	169.99 (18)	C7A'—C5A'—C6A'—C1A'	175.5 (3)
O2—S—C1—C6	38.1 (2)	C4A'—C5A'—C6A'—C1A'	51.3 (3)
N—S—C1—C6	−73.15 (19)	C1A'—N—C1B'—C2B'	40 (5)
C6—C1—C2—C3	−0.4 (4)	S—N—C1B'—C2B'	−28.9 (10)
S—C1—C2—C3	178.6 (2)	C1A'—N—C1B'—C6B'	−113 (6)
C1—C2—C3—C4	0.4 (4)	S—N—C1B'—C6B'	177.9 (6)
C2—C3—C4—C5	−0.2 (4)	N—C1B'—C2B'—C3B'	179.2 (8)
C2—C3—C4—Cl	−179.7 (2)	C6B'—C1B'—C2B'—C3B'	−30.1 (15)
C3—C4—C5—C6	0.0 (4)	C1B'—C2B'—C3B'—O3B'	−147.4 (12)
Cl—C4—C5—C6	179.5 (2)	C1B'—C2B'—C3B'—C4B'	26.1 (16)
C4—C5—C6—C1	0.0 (4)	O3B'—C3B'—C4B'—C5B'	135.8 (12)
C2—C1—C6—C5	0.2 (4)	C2B'—C3B'—C4B'—C5B'	−37.6 (15)
S—C1—C6—C5	−178.8 (2)	C3B'—C4B'—C5B'—C6B'	51.1 (13)
C1B'—N—C1A'—C2A'	−121 (6)	C3B'—C4B'—C5B'—C7B'	175.3 (11)
S—N—C1A'—C2A'	−6.9 (4)	C2B'—C1B'—C6B'—C5B'	43.2 (13)
C1B'—N—C1A'—C6A'	53 (6)	N—C1B'—C6B'—C5B'	−163.8 (7)
S—N—C1A'—C6A'	167.16 (19)	C4B'—C5B'—C6B'—C1B'	−51.6 (11)
N—C1A'—C2A'—C3A'	178.3 (3)	C7B'—C5B'—C6B'—C1B'	−175.9 (10)

### Hydrogen-bond geometry (Å, °)

**Table:**

*D*—H···*A*	*D*—H	H···*A*	*D*···*A*	*D*—H···*A*
N—H1N···O3B'^i^	0.83 (2)	1.91 (2)	2.729 (10)	166 (2)
N—H1N···O3A'^i^	0.83 (2)	1.95 (2)	2.777 (3)	171 (2)
C5—H5A···O2^ii^	0.93	2.53	3.337 (3)	145.

Symmetry codes: (i) *x*, −*y*+3/2, *z*+1/2; (ii) −*x*+2, *y*−1/2, −*z*+3/2.

### Table 2 Selected geometric parameters (Å, °) calculated with X-ray and DFT

**Table:**

Parameters	X-ray	DFT/B3LYP(3-21G**)
Cl—C4	1.7404 (12)	1.7556
S—O1	1.4262 (9)	1.4610
S—O2	1.4300 (9)	1.4613
S—N	1.6397 (7)	1.6831
S—C1	1.7675 (10)	1.7663
N—C1A'	1.4049 (11)	1.4055
O3A'—C3A'	1.2376 (14)	1.2415
C5A'—C7A'	1.5102 (18)	1.5477
O1—S—O2	120.12 (5)	123.44
O1—S—N	109.07 (4)	108.92
O2—S—N	104.34 (5)	103.61
O1—S—C1	108.40 (5)	107.79
O2—S—C1	108.31 (5)	108.21
N—S—C1	105.69 (4)	103.02
C6—C1—C2	120.93 (9)	121.18
C6—C1—S	118.79 (8)	120.04
C3—C4—C5	121.65 (11)	121.58
C3—C4—Cl	119.40 (10)	119.14
C5—C4—Cl	118.95 (9)	119.28
C2A'—C1A'—C6A'	122.58 (8)	122.53
C7A'—C5A'—C4A'	110.73 (11)	111.36
C1—S—N—C1A'	-67.84 (8)	76.42
N—S—C1—C6	-73.16 (8)	88.67
N—S—C1—C2	107.82 (8)	-92.03
S—N—C1A'—C2A'	-8.93 (14)	13.37
